# The Effects of Fruiting Positions on Cellulose Synthesis and Sucrose Metabolism during Cotton (*Gossypium hirsutum* L.) Fiber Development

**DOI:** 10.1371/journal.pone.0089476

**Published:** 2014-02-20

**Authors:** Yina Ma, Youhua Wang, Jingran Liu, Fengjuan Lv, Ji Chen, Zhiguo Zhou

**Affiliations:** Key Laboratory of Crop Growth Regulation, Ministry of Agriculture, Nanjing Agricultural University, Nanjing, Jiangsu Province, PR China; New Mexico State University, United States of America

## Abstract

Cotton (*Gossypium hirsutum* L.) boll positions on a fruiting branch vary in their contribution to yield and fiber quality. Fiber properties are dependent on deposition of cellulose in the fiber cell wall, but information about the enzymatic differences in sucrose metabolism between these fruiting positions is lacking. Therefore, two cotton cultivars with different sensitivities to low temperature were tested in 2010 and 2011 to quantify the effect of fruit positions (FPs) on fiber quality in relation to sucrose content, enzymatic activities and sucrose metabolism. The indices including sucrose content, sucrose transformation rate, cellulose content, and the activities of the key enzymes, sucrose phosphate synthase (SPS), acid invertase (AI) and sucrose synthase (SuSy) which inhibit cellulose synthesis and eventually affect fiber quality traits in cotton fiber, were determined. Results showed that as compared with those of FP1, cellulose content, sucrose content, and sucrose transformation rate of FP3 were all decreased, and the variations of cellulose content and sucrose transformation rate caused by FPs in Sumian 15 were larger than those in Kemian 1. Under FP effect, activities of SPS and AI in sucrose regulation were decreased, while SuSy activity in sucrose degradation was increased. The changes in activities of SuSy and SPS in response to FP effect displayed different and large change ranges between the two cultivars. These results indicate that restrained cellulose synthesis and sucrose metabolism in distal FPs are mainly attributed to the changes in the activities of these enzymes. The difference in fiber quality, cellulose synthesis and sucrose metabolism in response to FPs in fiber cells for the two cotton cultivars was mainly determined by the activities of both SuSy and SPS.

## Introduction

Cotton (*Gossypium hirsutum* L.) fiber is an important raw material for the textile industry. Thus, its yield and quality are the key criteria for cotton fiber value evaluation. The cotton plant has a prominent main stem and an indeterminate growth habit [1]. It has been shown that bolls at different fruiting positions (FPs) can produce different yield and fiber quality [2] [3].

Cotton yield is mainly determined by boll number and boll size [4]. Previous reports indicated that the position of the first node on a sympodial branch (FP1) contributed more to higher yield than the other FPs did on the same sympodial branch, and the relative contribution of FPs 1, 2, and 3 accounted for about 60%, 30% and 10% of the total yield of seed cotton, respectively [1] [2] [5] [6]. However, in some fields where cotton yield was high (i.e. 7657Kg·ha-1) in the Yangtze River Valley of China, the boll retention rate on the distal sites (e.g. FP3 and greater) reached as high as 58.8% [7]. Thus, the increased boll retention rate of distal FPs might be the key for improving fiber yield in the fields where cotton yield potential is high.

Fiber quality depends on complex interactions among the genetic and physiological factors. The effect of the growth environment on the genetic potential of a genotype modulates fiber properties to varying degrees [8] [9] [10]. For example, application of water or fertilizer and the inevitable seasonal shifts such as temperature, day length, and insolation all could realize the changes of genetic potential [11] [12] [13] [14]. Previous documents on fiber qualities under environmental factors were mainly concentrated on the adaxial fruiting positions (e.g. FP1 and FP2) [3] [15] [16] [17], but nothing has been conducted on fiber qualities of the distal's (e.g. FP3 and greater).

Up to 90% of mature cotton fiber is consisted of cellulose. Thus, the process of cotton fiber formation primarily is a process of cellulose synthesis [18]. Sucrose is the initial carbon source for cellulose synthesis and supplies the UDP-glucose (UDP-G) as the immediate substrate for cellulose polymerization [19] [20]. A number of enzymes are involved in the sucrose metabolism and can be classified into two types, sucrose-synthesis enzymes and sucrose-decomposition enzymes [21]. Sucrose phosphate synthase (SPS), which is consider as a major regulator in organs and tissues adapted to cold and drought stresses, regulates sucrose synthesis [22] [23]. Invertases, especially the acid invertase (AI) catalyse the irreversible hydrolysis of sucrose to glucose and fructose [24] [25]. In addition to AI, sucrose synthase (SuSy) can both degrade and synthesize sucrose, but its function in cotton fiber is primarily on reversible sucrose cleavage [18] [26].

Base on the background described above, we designed an experiment to study the response of related enzymes involved in sucrose metabolism at various stages of development in cotton fibers on different FPs, aiming at 1) finding the sensitive enzymes to FP effect in sucrose metabolism for the two cultivars; 2) clarifying the relationship between sucrose metabolism, cellulose synthesis and fiber qualities. Finally, elucidate the differences and their physiological mechanisms among fiber cells of the bolls at different FPs. This study could provide further supplement and extension for the research of fruiting positions and would be valuable for cotton cultivators to improve cotton yield and fiber quality by distal FPs.

## Materials and Methods

### Plant material and experimental design

Field experiments were conducted at the Pailou experimental station of the Nanjing Agricultural University, Nanjing (32°02′N and 118°50′E), Jiangsu (the Yangtze River Valley), China, in the Yangtze River Valley from 2010 to 2011. The soil at the experimental site was clayed, mixed, thermic, Typic alfisols (udalfs; FAO luvisol) in 20 cm depth of the soil profile, and the nutrient contents of soil before planting cotton contained 18.5 and 16.3 g·kg^−1^ organic matter, 1.1 and 1.0 g·kg^−1^ total N, 64.4 and 50.2 mg·kg^−1^ available N, 17.9 and 16.8 mg·kg^−1^ available P, and 102.3 and 96.4 mg·kg^−1^ available K. Cotton cultivars had different sensitivities to low temperature, 14 diverse cultivars which were widely grown in the Yangtze River Valley in China were studied by Wang et al. [27] with different mean daily temperature during fiber development period. Based on the variance of fiber strength, these cultivars were clustered into three groups as a temperature-sensitive group (typical for Sumian 15), a moderately sensitive group (typical for NuCOTN 33B) and a temperature-insensitive group (typical for Kemian 1). Bolls from different FPs in the same sympodial branches with different flowering dates, therefore, Kemian 1 (temperature-insensitive) and Sumian 15 (temperature-sensitive) were selected for this study. Cotton seeds were sown in a nursery bed on 25 April, and seedlings with three true leaves were transplanted to field. Each plot size was 5.6 m wide by 6 m long, with row spacing of 80 cm and interplant spacing of 25 cm. Three replications for each cultivar were assigned randomly in the field. The nitrogen fertilizer applied 40% before transplanting and 30%, 30% applied at first flowering and peak flowering for each cultivar, respectively. Furrow-irrigation was applied as needed to minimize the moisture stress during each season.

### Sampling and processing

Cotton flowers were labeled at anthesis with a tag listing the date at FPs 1 and 3 of the 7^th^ sympodial branches, respectively (hereafter FP1 and FP3). The 7^th^ sympodial branch is located in the middle of cotton canopy, which has better fiber quality than the bottom and upper branches do. It is usually used as the typical branch to research in document [28] [29] [30]. White flowers were tagged for each cultivar on the same day, no more than 3 days after the start of tagging, to ensure that the tagged flowers were of equivalently metabolic and developmental ages for each cultivar. The 17, 31, and 45 days post anthesis (DPA) are usually regarded as the key representative stages to study the physiological characteristics during cotton fiber development [18] [21] [31] [32]. Therefore, subsequently, the labeled boll samples (about 6–8 bolls in each cultivar) were collected at the 17^th^, 31^st^ and 45^th^ DPA, respectively. Cotton boll samples were harvested at 9∶00–11∶00 am, and fibers were excised from the bolls with a scalpel and were immediately put into liquid nitrogen for subsequent measurement. Tagged bolls (about 10–15 bolls) in each replications were hand harvested after bolls opened and ginned in individual groups according to each fruiting position.

### Fiber quality, cellulose content and sucrose content quantification

Ginned fiber from each group was sent to the Cotton Quality Supervision, Inspection, and Testing Center of China Ministry of Agriculture for quality analysis. Fiber quality including fiber upper-half mean length (UHML), uniformity index (UI), strength (ST), elongation (EL) and micronaire (MIC) of each lint sample was read with a high volume instrument (HVI). Sucrose and glucose were extracted by a modified method of Pettigrew [33]. The sucrose and glucose assay was conducted according to the method described by Hendrix [34]. Sucrose transformation rate was calculated according to Shu et al. [21]. Fibers were digested in an acetic-nitric reagent, and the cellulose content was measured with anthrone according to the method described by Updegraff [35].

### Enzymatic analyses

Enzyme extracts were prepared essentially as described by King *et al.* [36]. Soluble acid invertase (AI) was measured by incubation of 100 μl of extract with 1 M sucrose in 200 mM acetic acid-NaOH (pH 5.0) in a total volume of 2.5 ml [36]. Reactions were started by incubating at 30°C for 30 min. The reactions were stopped by adding 1 ml of 3,5-dinitrosalicylic acid (DNS), and then boiled for 5 min. Glucose content was determined by relating the spectrophotometer metrically at 540 nm. Sucrose synthase (SuSy) activity was assayed by measuring the level of fructose formed from the cleavage of sucrose [36]. Each reaction contained 20 mM Pipes–KOH (pH 6.5), l00 mM sucrose, 2 mM UDP, and 200 μl of extract in a total volume of 650 μl. Reactions were started by incubating at 30°C for 30 min. The reactions were stopped by adding 250 μl of 0.5 M Tricine–KOH (pH 8.3), and then boiled for 10 min. The amount of fructose in SuSy reactions was determined and calculated from a standard curve of fructose at 540 nm. Sucrose phosphate synthase (SPS) activity was assayed by measuring the synthesis of sucrose-6-P [23]. Each reaction contained 14 mM UDP-glucose, 50 mM fructose-6-P, 50 mM extraction buffer, 50 mM MgCl_2_ and 200 μl of extract in a total volume of 650 μl. The reaction was started by incubating the enzyme extract at 30°C for 30 min. The reaction was stopped by adding 100 μl of 2 N NaOH and by boiling for 10 min at 100°C to destroy unreacted hexoses and hexose phosphates. The 1 ml of 0.1% (w/v) resorcin in 95% (v/v) ethanol was added and then incubated at 80°C for 30 min. Sucrose-6-P content was calculated based on a standard curve measured at 480 nm. UDPG and F-6-P were purchased from Sigma-Aldrich.

### Weather data and data analysis

Weather data across the two-year study period were collected from an established local weather station (Nanjing Weather Station) located near the experimental site (about 10 m away from the field)(Table 1). Analysis of variance was conducted with SPSS statistic package Version 17.0 and the difference between mean values greater than the LSD (*P* = 0.05) was determined as significant. The coefficient of variation (CV, %) was calculated as the ratio of the standard deviation to the mean. According to Zhao et al. [37], cumulative photo-thermal index (*PTI*), which represents the effect of temperature and radiation during cotton fiber development period, was calculated by equation (Eq)(1).

**Table 1:** Mean daily temperature, mean daily maximum temperature, mean daily minimum temperature, mean diurnal temperature difference, total solar radiation and cumulative photo-thermal index during cotton fiber development period from flowering date to boll opening date on FP1 and FP3 of two cultivars during 2010–2011.

Years	Fruiting positions	Planting date	Flowering dates	Boll opening dates	Fiber development period	MDT^a^	MDTmax^b^	MDTmin^c^	MDTdif^d^	MDSR^e^	*PTI* f
	(FPs)	(dd-mm)	(dd-mm)	(dd-mm)	(d)	(°C)	(°C)	(°C)	(°C)	(MJ·m^−2^)	(MJ·m^−2^)
2010	FP1	25-Apr	29-Jul	12-Sep	46	29.0^g^	33.1	25.8	7.3	17.1	683.4
	FP3	25-Apr	11-Aug	27-Sep	48	26.6	30.6	23.7	6.9	14.3	549.9
					CV^h^, %	4.27	3.87	4.3	2.49	9.17	10.83
2011	FP1	25-Apr	27-Jul	14-Sep	50	26.7	30.7	24.0	6.7	14.2	630.5
	FP3	25-Apr	11-Aug	1-Oct	52	24.5	28.3	21.6	6.7	12.7	479.8
					CV, %	4.38	3.97	5.18	0.11	5.84	13.58

a MDT, mean daily temperature.

b MDTmax, mean daily maximum temperature.

c MDTmin, mean daily minimum temperature.

d MDTdif, mean diurnal temperature difference.

e MDSR, mean daily solar radiation.

f *PTI*, cumulative photo-thermal index during cotton fiber development period.

g Weather data were provided by Nanjing Weather Station, which was located nearby the experimental site.

h CV, coefficient of variation.



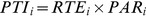
(1)where RTE refers to daily relative thermal effectiveness, according to the non-linear response curves of boll development to temperature [38], RTE_i_ is calculated by Eq. (2), and the relative temperature effect (RTE (*T*)) is calculated by Eq. (3). PAR is daily photosynthetically active radiation.



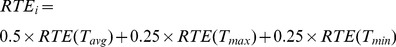
(2)

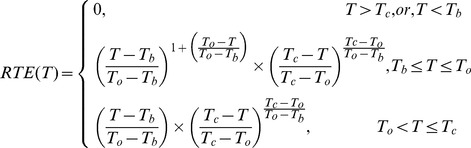
(3)where *T*_avg_, *T*_max_, and *T*_min_ refer to the daily average, maximum, and minimum air temperature, respectively. *T*_b_, *T*_o_, and *T*_c_ are the cardinal temperature values (base, optimum, and ceiling temperatures) for development. They were retained as 15, 30, and 35°C, respectively.

## Results and Discussion

### Environmental conditions

The flowering date and cotton fiber development period (CFDP), i.e. from flowering date to boll opening date, were affected by the boll positions (Table 1). Generally, at the same planting date, the flowering date of bolls on FP1 was about two weeks earlier than that on FP3 in the same sympodial branches [5]. But the CFDPs differed by only one or two-days between FP1 and FP3. Thus, the differences between FP1 and FP3 were primarily due to the changes in environmental conditions at the various flowering dates. The coefficients of variance of cumulative photo-thermal index (*PTI*) during CFDP were higher than those of mean daily temperature (MDT), mean daily maximum (MDT max), mean daily minimum (MDT min), mean diurnal temperature difference (MDTdif), and mean daily solar radiation (MDSR) in both 2010 and 2011 (Table 1). This indicated that the difference in environmental conditions during the fiber development period for different FPs was primarily on *PTI*. The *PTI* of FP3 was 19.5–23.9% lower than that of FP1.

### Fiber quality in cotton fiber

A number of the fiber quality traits including UHML, ST and MIC were significantly affected by FPs in both 2010 and 2011 (Table 2). Compared to those of FP1, UHML, ST and MIC of FP3 were all decreased in both Kemian 1 and Sumian 15. However, EL and UI were not affected by FPs in any year. When both years were combined and fiber qualities were analyzed, there was no significant effect of year and interaction of years × cultivars, years × fruiting positions and cultivars × fruiting positions for all fiber qualities. However, there were highly significant differences (*P<*0.01) in UHML and MIC, and significant differences (*P<*0.05) in EL and ST between cultivars but there was no significant difference (*P>*0.05) of cultivars for UI. The two cultivars, Kemian 1 and Sumian 15, had different ranges of UHML, ST and MIC between FP1 and FP3. The CVs of UHML, ST and MIC for Sumian 15 was greater than that of Kemian 1. These results indicated that cotton fiber quality was more easily affected by FPs in Sumian 15 than in Kemian 1.

**Table 2:** Fiber properties of field-grown cotton on FP1 and FP3 of two cultivars during 2010–2011.

Years	Cultivars	Fruiting positions	UHML^a^	UI^b^	MIC^c^	EL^d^	ST^e^
		(FPs)	(mm)	(%)		(%)	(cN tex^−1^)
2010	Kemian 1	FP1	30.5a^f^	84.3a	4.9a	6.4a	30.3a
		FP3	29.6b	84.2a	4.7b	6.4a	29.3b
		CV^g^,%	1.58	0.11	2.77	0.39	1.78
	Sumian 15	FP1	29.8a	84.0a	4.7a	6.2a	29.6a
		FP3	28.7b	83.5a	4.4b	6.2a	28.4b
		CV,%	1.77	0.34	4.12	0.28	2.07
2011	Kemian 1	FP1	30.7a	85.1a	4.9a	6.3a	31.4a
		FP3	29.8b	84.9a	4.6b	6.2a	29.9b
		CV,%	1.38	0.10	3.31	0.34	2.42
	Sumian 15	FP1	30.0a	84.6a	4.7a	6.1a	30.5a
		FP3	28.8b	84.3a	4.3b	6.1a	28.7b
		CV,%	2.04	0.18	4.36	0.25	3.04
Source of variation							
Years			ns h	ns	ns	ns	ns
Cultivars			** h	ns	**	* h	*
Fruiting positions			**	ns	**	ns	**
Years × Cultivars			ns	ns	ns	ns	ns
Years × Fruiting positions			ns	ns	ns	ns	ns
Cultivars × Fruiting positions			ns	ns	ns	ns	ns
Years × Cultivars × Fruiting positions			ns	ns	ns	ns	ns

a UHML, fiber upper-half mean length.

b UI, uniformity index.

c MIC, micronaire value.

d EL, elongation percentage.

e ST, fiber strength.

f Values followed by a different letter between fruiting positions are significantly different at *P* = 0.05 probability level. Each value represents the mean of three replications.

g CV, coefficient of variation.

h * and ** indicate significant differences at P≤0.05 and 0.01 probability levels, respectively. ns, not significant (P≥0.05).

Previous studies have documented that fiber length is largely dependent on genetic factors, while fiber maturity properties, which are dependent on deposition of photosynthates in the fiber cell wall, are more sensitive to changes in the growth environment [9] [39]. However, several studies have indicated that fiber length could be significantly affected by cool temperature or planting dates [10] [16] [40] [41]. Thus, fiber qualities such as length, strength, and micronaire etc. are probably affected by the changing growth environment. Previous reports about FP effects on fiber quality mainly concentrated on longitudinal direction of the main stem [42] [43], but few studies paid attentions to the horizontal direction. In term of the horizontal direction, Heitholt [44] found that FP had no effect on fiber strength, maturity and micronaire values, whereas Pettigrew [15] documented the opposite results. In addition, Davidonis et al. [3] indicated that boll position of the horizontal direction could affect the fiber quality indices such as fiber length. Hence, FP effects on fiber quality traits are not consistent, and the possible reason for this disparity might be due to the differences in cultivars and the various environment conditions during the CFDP. Our results showed that fiber length, strength and micronaire values from FP1 were significantly higher (*P<*0.05) than those from FP3 (Table 2), which were consistent with those reported by Pettigrew [15] on fiber strength and by Davidonis [3] on fiber length and micronaire values. Since the difference in the effects caused by environmental conditions during the CFDP for different FPs was primarily on *PTI* (Table 1), thus, our results have indicated that the shorter and weaker fibers from FP3 are correlated with its lower *PTI*.

### Cellulose content and sucrose content in cotton fiber

**Figure 1:**
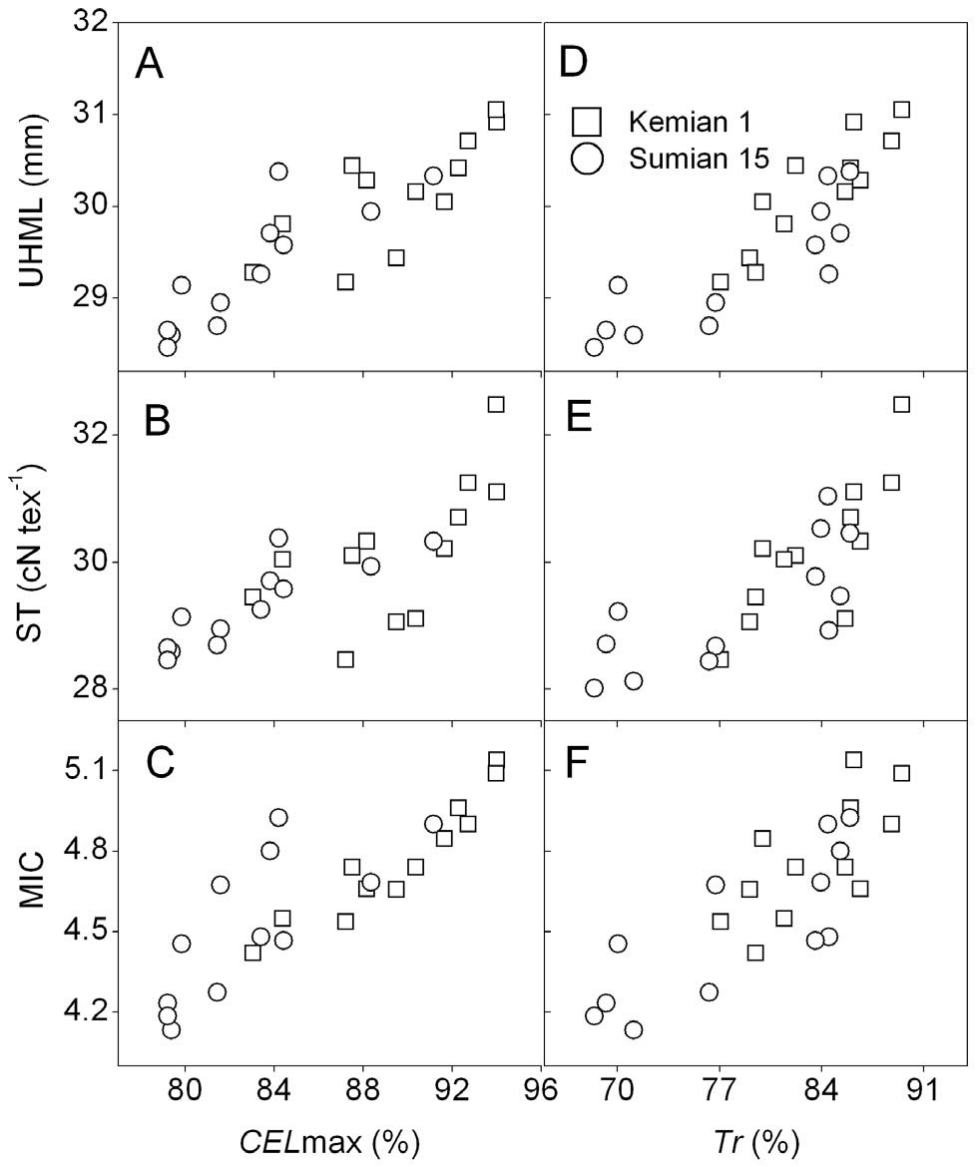
Correlation coefficients among parameters in fibers on FP1 and FP3 of two cultivars during 2010–2011. The coefficient between UHM and *CEL*max (A), ST and *CEL*max (B), MIC and *CEL*max (C), UHM and *Tr* (D), ST and *Tr* (E), and MIC and *Tr* (F) in fibers on FP1 and FP3 of two cultivars. UHML-fiber upper-half mean length, MIC-micronaire value, ST-fiber strength, *CEL*max-maximum cellulose content, *Tr*-sucrose transformation rate. * and **, significant differences at *P* = 0.01 and *P* = 0.05 probability levels, respectively. n = 12, *R*_0.05_ = 0.576, *R*_0.01_ = 0.707.

Cellulose content in cotton fibers increased from 17 DPA (Table 3). In both cultivars Kemian 1 and Sumian 15, the cellulose content at FP1 was significantly higher (*P<*0.05) than that at FP3. Finally, at the mature stage, the cellulose content of the fibers from FP1 was 3–10% higher than that from FP3. Cotton fiber quality is predominantly determined by the process of cellulose synthesis [31] [45]. And environmental factors such as cool temperature can alter fiber strength by reducing cellulose content within secondary cell walls [46] [47]. Comparing with that of FP1, FP3 had a lower *PTI* restrained cellulose synthesis. The correlation coefficient of cellulose content with the key fiber properties indicated that fiber length was positively correlated with ultimate cellulose content, and so did fiber strength and micronaire values (Fig. 1A, Fig. 1B and Fig. 1C). The coefficients for the correlations between fiber length and maximum cellulose content, fiber strength and maximum cellulose content, and fiber micronaire values and maximum cellulose content were 0.760^**^, 0.634^*^ and 0.938^**^ (^**^ *P<*0.01, ^*^ *P<*0.05), respectively, in Kemian 1, and were 0.857^**^, 0.894^**^ and 0.758^**^ (^**^ *P<*0.01), respectively, in Sumian 15. These results indicate that fiber quality traits are correlated with the maximum cellulose content and that these correlations are influenced by the effects of FPs.

**Table 3:** Cellulose content, sucrose content and sucrose transformation rate in cotton fibers on FP1 and FP3 of two cultivars during 2010–2011.

Years	Cultivars	Fruiting positions	Cellulose content (%)			Sucrose content (mg g^−1^ DW)			*Tr* a
		(FPs)	17DPA^b^	31DPA	45DPA	17DPA	31DPA	45DPA	(%)
2010	Kemian 1	FP1	46.88a^c^	74.55a	92.22a	11.91a	6.68a	1.67a	85.93a
		FP3	38.79b	68.80b	89.46b	9.80b	6.17b	2.09a	78.69b
	Sumian15	FP1	43.18a	70.25a	83.82a	10.70a	6.21a	1.58a	85.25a
		FP3	34.91b	63.41b	80.81b	8.19b	5.47b	2.08a	74.71b
2011	Kemian 1	FP1	41.93a	74.16a	91.62a	12.70a	6.40a	1.49a	88.31a
		FP3	30.85b	66.20b	84.98b	9.95b	5.52b	1.89b	81.04b
	Sumian15	FP1	40.82a	71.04a	87.99a	11.79a	5.75a	1.89a	84.01a
		FP3	27.38b	62.20b	79.43b	8.19b	4.59b	2.52b	69.23b

a *Tr*, sucrose transformation rate.

b DPA, days post anthesis.

c Values followed by a different letter between fruiting positions are significantly different at *P* = 0.05 probability level. Each value represents the mean of three replications.

During the cotton fiber development period, sucrose content in cotton fibers declined from 17 DPA to 45 DPA at both FP1 and FP3 (Table 3). Compared to that of FP3, sucrose content in bolls on FP1 was significantly higher (*P<*0.05) in both 17 DPA and 31 DPA, but was lower at 45 DPA. This indicated that the decreased rate of sucrose content in bolls on FP3 was slower than that on FP1. According to Shu et al. [21], we used the maximum sucrose content and minimum sucrose content in cotton fibers to calculate the sucrose transformation rate, which reflected the sucrose transformation capacity during cotton fiber development in our experiment. The result showed that the FP effect significantly decreased the sucrose transformation rate (*P<*0.05) in both cultivars (Table 3). During cotton fiber development, the correlations of environment factors with maximum/minimum sucrose content and sucrose transformation rate were analyzed (Table 4). Sucrose transformation rate in cotton fiber were positively correlated to *PTI* (*P<*0.05), but MDT, MDTmin, MDTmax, MDTdif and MDSR were not significantly related to it (*P>*0.05). This indicates that sucrose metabolism of fiber during CFDP of FPs are determined primarily by *PTI*.

**Table 4:** Correlation coefficient of environmental factors during cotton fiber development period with maximum/minimum sucrose content and sucrose transformation rate in cotton fibers on FP1 and FP3 of two cultivars during 2010–2011.

Correlation with	MDT	MDTmax	MDTmin	MDTdif	MDSR	*PTI*
*SUC*max^a^	0.512	0.514	0.558	0.168	0.446	0.740* d
*SUC*min^b^	−0.666	−0.667	−0.690	−0.423	−0.625	−0.778*
*Tr* c	0.625	0.626	0.658	0.336	0.572	0.781*

a *SUC*max, maximum sucrose content.

b *SUC*min, minimum sucrose content.

c *Tr*, sucrose transformation rate.

d n =  8, *R*_0.05_ = 0.707, *R*_0.01_ = 0.834. *, significant differences at *P* = 0.05 probability level.

**Figure 2:**
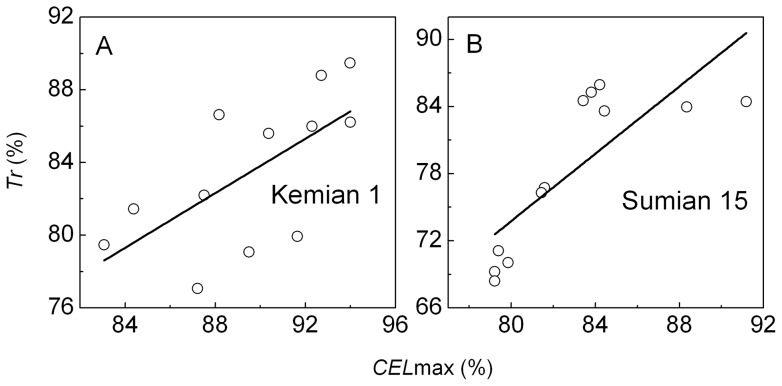
Correlation coefficients between cellulose content and sucrose transformation rate in fibers during 2010–2011. The coefficient between *CEL*max and *Tr* in fibers on FP1 and FP3 of Kemian 1 (A), *CEL*max and *Tr* in fibers on FP1 and FP3 of Sumian 15 (B). *CEL*max-maximum cellulose content, *Tr-*sucrose transformation rate. * and **, significant differences at *P* = 0.01 and *P* = 0.05 probability levels, respectively. n = 12, *R*_0.05_ = 0.576, *R*_0.01_ = 0.707.

The basic mechanisms regulating cellulose synthesis in different plant species are believed to be similar [18] [19] [20]. Sucrose metabolism is the pivotal process for cellulose synthesis and is sensitive to environment conditions. In many plants, the sucrose metabolism was restrained when they were subjected to low temperature [48] [49]. In our results, there was a positive correlation between maximum cellulose content and sucrose transformation rate in fibers when subjected to FP effect (*P<*0.05) (Fig. 2A and Fig. 2B). And the coefficient of this correlations was 0.653^*^ in Kemian 1 (^*^ *P<*0.05) and 0.801^**^ in Sumian 15 (^**^ *P<*0.01), respectively. This analysis indicates that fiber development and cellulose synthesis are determined primarily by sucrose metabolism as it is influenced by the FP effect.

Moreover, the coefficients for the correlation between sucrose transformation rate with the key fiber properties indicated that fiber length was positively correlated with sucrose transformation rate, and so did the fiber strength and micronaire values (Fig. 1D, Fig. 1E and Fig. 1F). The coefficients for the correlations between fiber length and sucrose transformation rate, fiber strength and sucrose transformation rate, and fiber micronaire values and sucrose transformation rate were 0.893^**^, 0.808^**^ and 0.719^**^ (^**^ *P<*0.01) in Kemian 1, were 0.850^**^, 0.750^**^ and 0.798^**^ in Sumian 15 (^**^ *P<*0.01), respectively. Based upon these results we indicate that lower *PTI*, which is caused by FP effect, affect the sucrose transformation process from sucrose to cellulose to weaken the fiber qualities.

The coefficients of variance of the sucrose transformation rate between FP1 and FP3 were different in the two cultivars. In Sumian 15, the CVs of the sucrose transformation rate were 6.59% in 2010 and 9.63% in 2011, respectively, and in Kemian 1, they were 4.40% in 2010 and 4.29% in 2011, respectively. These results indicated that FPs had more effects on cotton fiber sucrose metabolism in cultivar Sumian 15 than it did in cultivar Kemian 1.

### Changes in activities of sucrose metabolism enzymes

**Figure 3:**
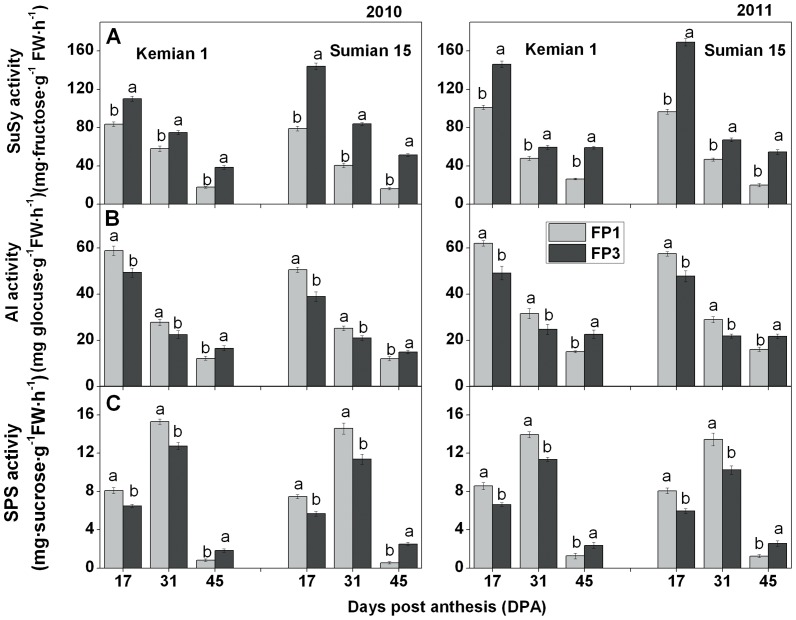
Changes of enzymes activities in fibers on FP1 and FP3 of two cultivars during 2010–2011. The activities of SuSy (A), AI (B) and SPS (C) in fibers on FP1 and FP3 of two cultivars. SuSy-sucrose sythase, AI-acid invatse and SPS-sucrose phosphate sythase. Values followed by a different letter between fruiting positions are significantly different at *P* = 0.05 probability level. Each value represents the mean of three replications.

It has been documented that SuSy, AI, and SPS are the critical enzymes involved in sucrose metabolism [50] [51]. In our study, we observed that these enzymes were affected by the FP effect, which might be the reason why sucrose transformation and cellulose synthesis in cotton fiber were restrained on distal FPs (e.g. FP3). The total SuSy activity including the activities of both soluble SuSy (S-SuSy) and the membrane-associated SuSy(M-SuSy) in cotton fibers declined from 17 DPA to 45 DPA (Fig. 3A). FP effect enhanced SuSy activity in cotton fiber, and changes in response to FP effect were affected by cotton fiber developmental age. In both Kemian 1 and Sumian 15, SuSy activity at FP3 was significantly higher (*P<*0.05) than that on FP1, and the largest variation was seen at 45 DPA (Table 5). SuSy, especially M-SuSy, is the critical partner in high-rate secondary-wall cellulose synthesis [52]. The suppression of SuSy activity by 70% or more in the ovule epidermis led to a fiberless phenotype and it is supposed that the increase or decrease in SuSy activity is associated with the increment or decrement of cellulose synthesis [53]. However, in this study, we observed that this high level of SuSy activity at FP3 did not enhance cellulose synthesis. This may be due to the reason that while SuSy contained both M-SuSy and S-SuSy in cotton fiber, a part of M-SuSy became S-SuSy under stress [18] [50]. In this study on distal FPs (like FP3), the increased SuSy activity was likely due to the enhanced S-SuSy activity, which supplied UDP-glucose for general metabolic needs rather than having a major regulatory role in partitioning of carbon to cellulose [18] [52] [54] [55].

**Table 5:** Comparisons of effect indices and coefficients of variations on the activities of sucrose metabolism enzymes in cotton fibers on FP1 and FP3 of two cultivars during 2010–2011.

Years	DPA^a^	SuSy activity		SPS activity		AI activity	
		Kemian 1	Sumian15	Kemian 1	Sumian15	Kemian 1	Sumian15
EI^b^, (%)							
2010	17	−31.75	−82.30	19.68	23.69	16.11	23.10
	31	−28.92	−107.51	16.58	21.93	19.08	16.76
	45	−116.47	−215.25	−123.89	−346.97	−34.84	−24.55
2011	17	−44.62	−75.11	22.53	26.08	20.71	16.85
	31	−23.68	−43.73	18.50	23.72	21.73	24.72
	45	−123.88	−173.71	−86.08	−103.44	−50.6	−34.49
CV^c^, (%)							
2010	17	13.70	29.15	10.92	13.43	8.76	13.06
	31	12.63	34.96	9.04	12.32	10.54	9.15
	45	36.80	51.84	38.25	63.43	14.83	10.93
2011	17	18.24	27.30	12.69	15.00	11.55	9.20
	31	10.59	17.94	10.19	13.46	12.19	14.10
	45	38.25	46.48	30.09	34.09	20.19	14.71

a DPA, day post anthesis.

b EI, the effect indices values, which were calculated as EI_X_ =  (X_FP1_-X_FP3_)/X_FP1_^*^100%. If EI>0, it is a decrease variation in FP3 compared with FP1. If EI<0, it is an increase variation in FP3 compared with FP1.

c CV, coefficient of variation (%).

The activity of AI decreased with the distal FPs in the immature fiber stage (17 DPA and 31 DPA), but increased at 45 DPA (Fig. 3B). Usually, isoforms of invertase in the cell wall and vacuole is AI, which may act as the major player in response to biotic and abiotic stresses [56] [57] [58]. For example, AI preferred to direct hydrolysis of sucrose to provide energy for maintaining fiber and winter oat development under cool temperature and cold hardening [59] [60]. In addition, AI affects the size of intracellular sucrose pools, but it appears to be mostly involved in regulating plant processes such as phloem unloading [61]. Therefore, our results indicate that this decreased AI activity by FPs effect affects the source-to-sink unloading of sucrose and ultimately weakens sink strength.

SPS is a key enzyme for sucrose synthesis and regulates sucrose accumulation [27]. The changing trends of SPS were similar in all cotton fibers, the peak values occurred at 31 DPA (Fig. 3C). Compared to those of FP1, the peak activity values were lower and the activity declined slowly at FP3. The largest differences in decreased extent of the activity between FP1 and FP3 were observed at 17 DPA (Table 5). Haigler et al. [62] indicated that under controlled environmental conditions, in cotton plants over-expressing SPS, sucrose synthesis and fiber quality was enhanced. Therefore, we presumed that the decreased SPS activity in cotton fiber on FP3 would hinder the flux of sucrose from glucose, which reduced the sucrose synthesis and cellulose synthesis.

In addition, according to Table 5, we found that the CVs of the activities of SuSy, SPS and AI at 45 DPA were all higher than those at 17 and 31 DPA. Moreover, the CVs of the activities of SuSy and SPS were much higher than that of AI activity. These results showed that the activities of SuSy and SPS were easily affected by FPs involved in sucrose metabolism. Besides, the activities of SuSy and SPS in the two cultivars had different sensitivities to FP effect, with higher CV in Sumian 15 than in Kemian 1, indicating that they are more sensitive to FP effect in Sumian 15 (Table 5). These indicated that the differences in SuSy and SPS activities in two cultivars in response to FP effect might be the reason for them to possess different sensitivities to boll positions for cellulose synthesis and sucrose metabolism.

This study revealed that both cellulose synthesis and sucrose metabolism were restrained by fruiting position effects. Hence, alternative strategies for alleviating the unfavorable effects of FPs need to be considered. Previous researches on the removal from specific fruiting positions in cotton indicated that fiber qualities on the second sympodial fruiting positions could be increased if the nutrition was sufficient [15] [44]. Nitrogen, phosphorus and potassium fertilization, planting density and abscisic acid may allow the crop to compensate partially for the potential yield losses and increase plant tolerance against abiotic stress such as cool temperature and low irradiance [12] [16] [37] [41] [63]. Therefore, elucidating the different mechanisms among different FPs in cotton fibers and improving the quality from distal positions are urgently needed. In this study, we found that FP effect restrained cellulose synthesis and sucrose utilization, and reduced the activities of the key enzymes involved in sucrose metabolism. Meanwhile, the examination of the effect of FPs on fiber development for two cultivars revealed that Sumian 15 was more sensitive to the FP effect than Kemian 1 was.

## Conclusions

In this study, we observed that FP effect affected cotton fiber development was primarily on cumulative photo-thermal index. Fiber length, fiber strength, and micronaire values, which are the critical fiber properties in textile processing, were all sensitive to FP effect. Highly correlated coefficients between these critical fiber quality traits and cellulose content revealed the close relationship between them under FP effect. Since sucrose metabolism is the pivotal process for cellulose synthesis, correlations between cellulose content and sucrose transformation rate of FP effect were analyzed and these coefficients were found to be highly significant. These results have indicated that sucrose metabolism determines cellulose synthesis under FP effect and both SuSy and SPS are the critical enzymes involved in sucrose metabolism and they also vary in cotton fibers on FP3 as compared to that on FP1. These variations indicate a potentially important reason for the decrease in sucrose and cellulose synthesis.
